# Pilot study of a novel lumen‐apposing metal stent for endoscopic ultrasound‐guided procedures in porcine models

**DOI:** 10.1002/deo2.70084

**Published:** 2025-03-23

**Authors:** Benedetto Mangiavillano, Gianluca Franchellucci, Francesco Auriemma, Daryl Ramai, Alberto Larghi, Danilo Paduano, Diletta De Deo, Federica Calabrese, Carmine Gentile, Matteo Fiacca, Antonio Facciorusso, Alessandro Repici

**Affiliations:** ^1^ Gastrointestinal Endoscopy Unit Humanitas ‐ Mater Domini Castellanza Italy; ^2^ Department of Biomedical Sciences Humanitas University Milan Italy; ^3^ Division of Gastroenterology and Digestive Endoscopy Humanitas Research Hospital‐IRCCS Rozzano Italy; ^4^ Division of Gastroenterology and Hepatology The University of Utah School of Medicine Salt Lake City Utah USA; ^5^ Digestive Endoscopy Unit, Fondazione Policlinico Universitario A. Gemelli IRCCS Università Cattolica del Sacro Cuore Rome Italy; ^6^ Department of Medical Sciences, Gastroenterology Unit University of Foggia Foggia Italy

**Keywords:** endoscopic ultrasound, EUS‐GBD, EUS‐GE, Hot‐Spaxus, lumen‐apposing metal stents

## Abstract

Lumen‐apposing metal stents have expanded the therapeutic potential of interventional endoscopic ultrasound (EUS). The Hot‐Spaxus (Taewoong Medical Co., Ltd.), the second most commonly utilized lumen‐apposing metal stent, requires two operators for its release which has been considered a limitation compared to other lumen‐apposing metal stents. We aimed to test the feasibility and the technical success of a newly available version of the Hot‐Spaxus stent equipped with an innovative handle delivery system for EUS‐guided interventional procedures. We conducted a pilot study using porcine models. The novel Hot‐Spaxus 2 was tested by performing four EUS‐guided procedures including four EUS‐guided gallbladder drainage and 12 EUS‐guided gastrojejunostomy) procedures. Technical success was reported in 100% of cases. The mean procedure time for EUS‐guided gatrojejunostomyJ and EUS‐guided gallbladder drainage was 23.85 min (standard deviation 3.41) and 16.15 min (standard deviation 2.72), respectively. The distal and proximal flanges were safely released by the endosonographer without any complications. No adverse events were reported. In conclusion, the novel Hot‐Spaxus 2 stent may represent an improvement compared to the prior Spaxus model. Unlike its predecessor, this newly designed stent eliminates the need for two endoscopists and can be deployed by a single operator. Further human studies are necessary to validate its clinical effectiveness.

## INTRODUCTION

Lumen‐apposing metal stents (LAMSs) allow the creation of stable anastomoses between the gastrointestinal (GI) tract and other organs or cavities. As a result, this has significantly expanded the role of interventional endoscopic ultrasound (EUS) over the past two decades.^1^, ^2^, ^3^, ^4^ LAMSs have been successfully used for pancreatic fluid collection drainage, creation of cholecystogastrostomy, cholecystoduodenostomy, or EUS‐guided choledocoduodenostomy (EUS‐CDS), and EUS‐guided gastrojejunostomy (EUS‐GJ).^5^ EUS‐CDS has been proven to be superior to percutaneous drainage in patients with distal malignant biliary obstruction after failed endoscopic retrograde cholangiopancreatography and appears to be as effective as endoscopic retrograde cholangiopancreatography when used as a first‐line therapeutic modality.^6^

Furthermore, EUS‐GJ will probably soon replace duodenal stenting and surgery for the treatment of patients with malignant gastric outlet obstruction when life expectancy is more than 3 months,^7^, ^8^ Finally, recent meta‐analyses and a randomized controlled trial have shown that gallbladder drainage using LAMS in patients with acute cholecystitis and high surgical risk is linked to significantly fewer adverse events (AEs), re‐interventions, and recurrent acute cholecystitis compared to the percutaneous route,^9^, ^10^

The first LAMS, the Axios stent (Boston Scientific Corp.), was released in 2011. A second LAMS (Spaxus; Taewoong Medical Co.) became available in 2016 and subsequently incorporated electrocautery capabilities on the tip (Hot‐Spaxus; Taewoong Medical). The Hot‐Spaxus has been widely utilized,^3^, ^11^, ^12^, ^13^ with high technical and clinical success rates.^5^, ^12^ In contrast to the Hot‐Axios, this LAMS lacks a single‐operator stent delivery system. To overcome these limitations, a second‐generation Hot‐Spaxus stent, known as Hot‐Spaxus 2, features a handle capable of stent release by a single operator along with new sizes and lengths.

We performed a pilot study using porcine models to test the technical feasibility of the new LAMS prototype, before introducing it into the market and clinical practice.

### Procedure

Four pigs were used to test the new Hot‐Spaxus 2. This study was conducted in adherence to the ARRIVE guidelines.^14^ The institutional review board of Humanitas Mater Domini approved the study (no 04/2025 HMD).

#### Improvement of Hot‐Spaxus 2 versus Hot‐Spaxus

**FIGURE 1:**
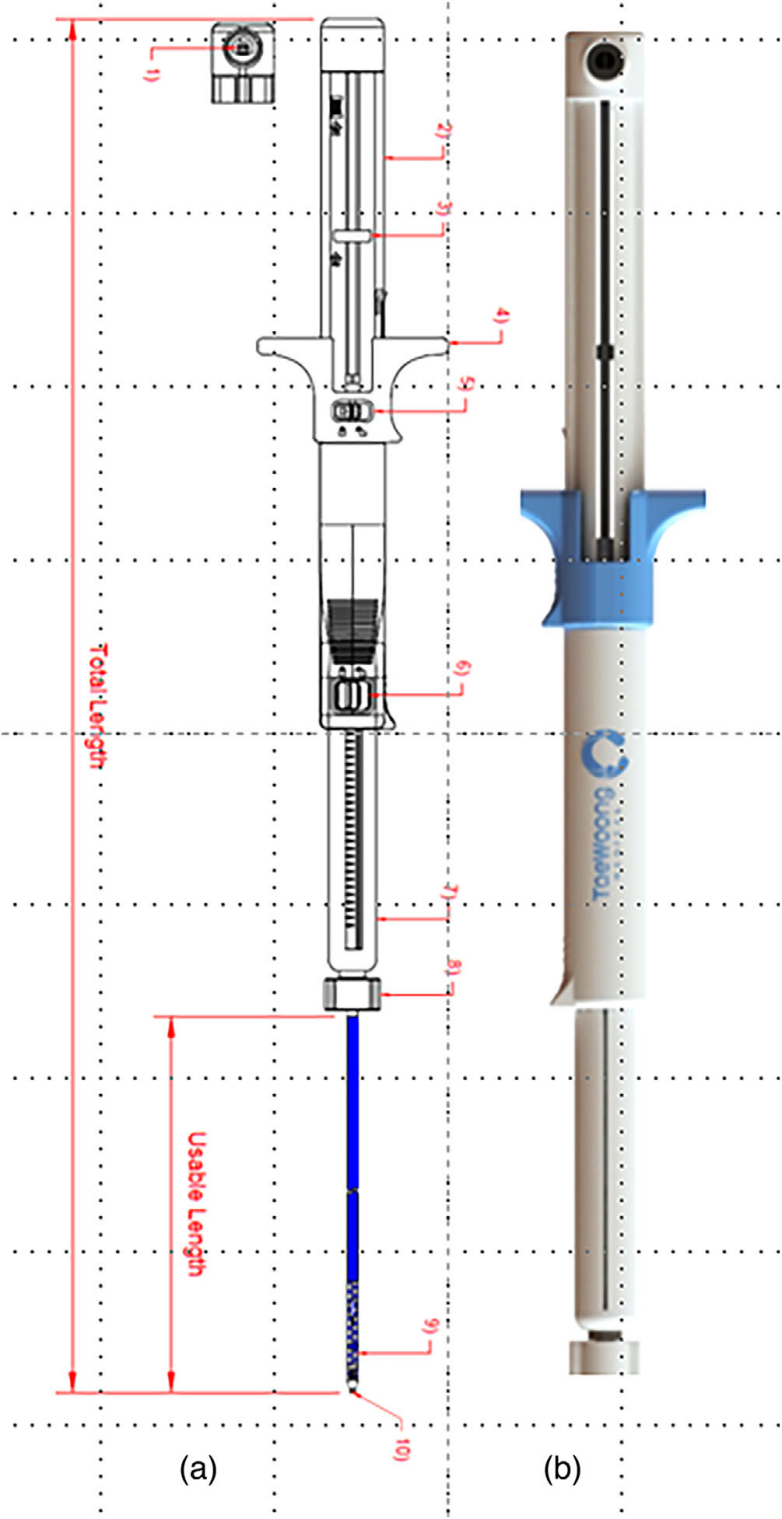
(a) Graphical description of Hot Spaxus 2 Handle. (b) Hot Spaxus 2 HandleGraphical description of the new Hot Spaxus Handle of the new Hot‐Spaxus 2 handle (one plug for connecting the electrosurgical unit, two main catheter control, three pins indicating half deployment markers, four stent deployment grip, five stent deployment grip controls, six main catheter control locks, seven luer lock covers, luer lock case, nine Hot‐Spaxus stent preloaded, and 10 holes for guide‐wire).

The Hot‐Spaxus 2 stent boasts two significant improvements. Firstly, a deployment handle system (Figure 1) that offers a double level of safety. Secondly, stent diameters and sizes have been updated as shown in Table 1. The stent's structure is crafted from nitinol and features a full silicone covering. The flange has a flexible structure that allows it to conform to the surface of the cavity.

**TABLE 1:** Length and diameter of LAMS Hot‐Spaxus 2.

Hot Spaxus stent new handle				
	Stent		Delivery	
Model	Body diameter (mm)	Length (mm)	Profile (Fr)	Usable Length (mm)
HSS0601FW‐N	6	10	8.5	1,380
HSS0801.2FW‐N	8	12	8.5	
HSS0802FW‐N	8	20	10	
HSS1002FW‐N	10	20	10	
HSS1601.5FW‐N	16	15	10	
HSS1602FW‐N	16	20	10	
HSS1801.5FW‐N	18	15	10.5	
HSS2001.5FW‐N	20	15	10.5	

The new handle includes a main catheter control, an operating knob to advance and retract the outer sheath for cauterization, an electrosurgical unit plug, a pin indicating stent half‐deployment mark, a stent‐deployment grip, a stent deployment lock to lock and unlock the stent after distal flange deployment, a main catheter control lock, a luer lock case for device engagement on the operative channel of the EUS scope, and a central channel that accomodates a 0.035″ guidewire (Figure 1).

#### Stent deployment preparation

**FIGURE 2:**
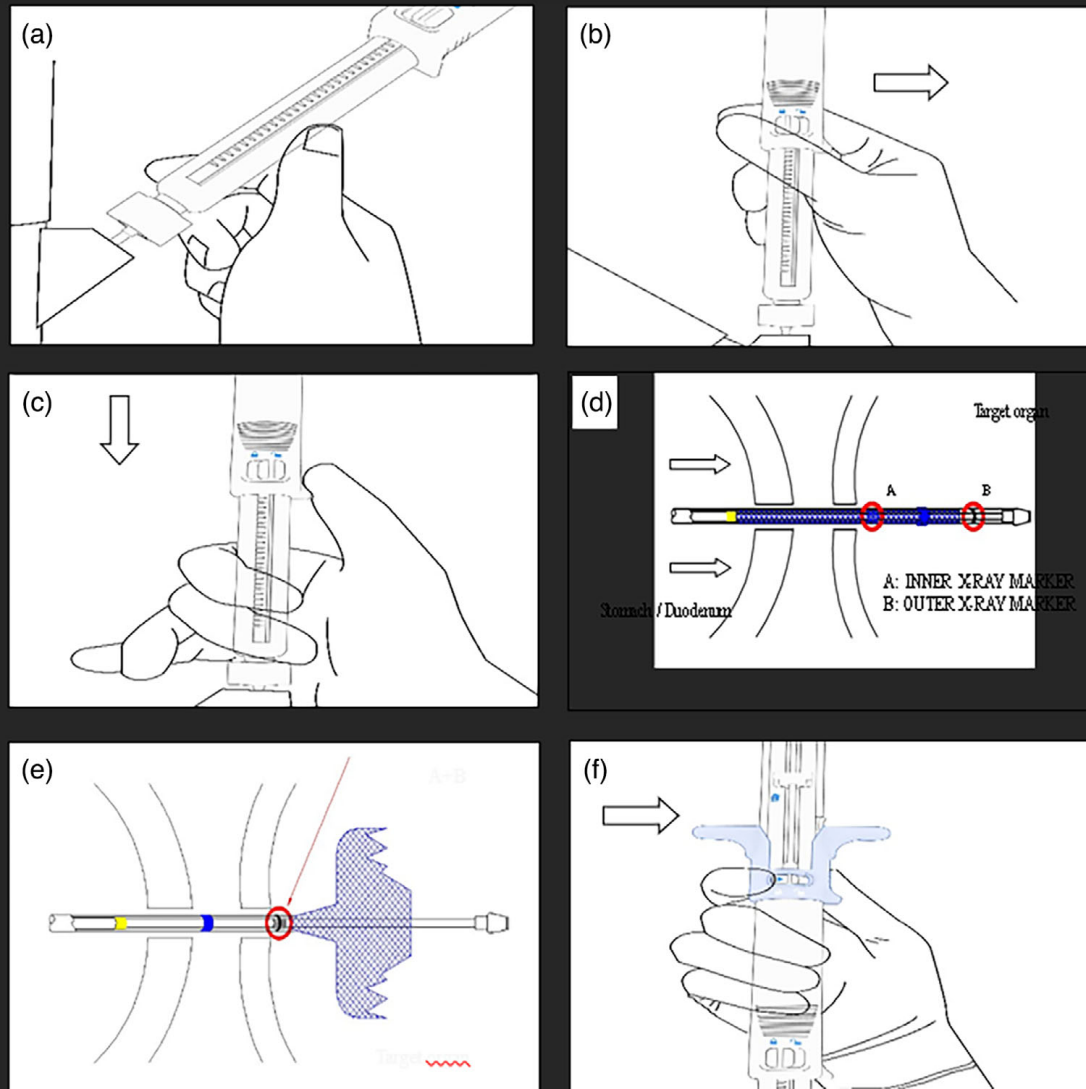
Graphic representation of steps involved in stent preparation and deployment Graphic representation of steps involved in stent preparation and deployment Graphic representation of handle. (a) Align the handle with the endoscope and attach it by rotating the luer lock fitting clockwise while keeping the handle stationary and aligned with the endoscope. (b) Push the main catheter lock to the right to unlock the catheter and to the left to relock it. (c) With the main catheter control unlocked, carefully advance the main catheter control (in the direction indicated by the arrow) so that the distal end of the catheter moves towards the gastrointestinal (GI) tract adjacent to the target structure. (d) Catheter marker “A” is then advanced into the target organ. (e) Distal flange opening into appropriate positioning. The stent deployment grip instantly locks into position with a “click” sound, and it cannot be moved out from position. (f) Push the stent deployment grip lock to the right to unlock the stent deployment grip.

The device must be inserted into the working channel of the EUS scope and advanced until the luer lock is aligned to the working channel fitting. Once the entire device fits the working channel of the echoendoscope, the luer lock should be rotated clockwise to secure the delivery system handle to the EUS operative channel (Figure 2a).

With the generator powered off, the stent delivery must be connected to the generator in monopolar current. Only pure‐cut settings should be used (80–120 W, 400–500 Vp).

Under EUS‐guided visualization, the position for stent delivery should be reconfirmed when the tip is outside of the scope for some millimeters. After unlocking the main catheter lock (Figure 2b), the device should be advanced by the handle into the target structure (Figure 2c).

**FIGURE 3:**
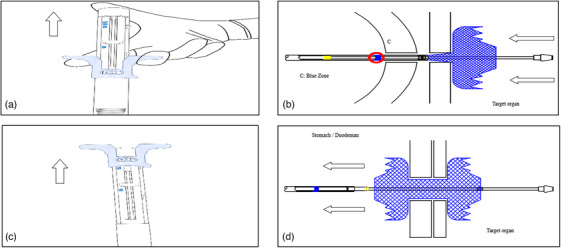
Stent releasing graphical display. (a) Retract the stent deployment grip to release the stent's first flange. (b) Pull the stent deployment grip all the way to deploy the proximal flare of the stent. (c) Deploy the stent deployment grip in the arrow direction points to the second flare of the stent. (d) The second flare is deployed and connects both walls together.

Under fluoroscopy and EUS guidance, the inner X‐ray marker (‘A’ of Figure 2d) should pass through the wall of the target structure. After the entrance, The main catheter should be locked to ensure that the delivery catheter will not move during the deployment of the stent flare. Slowly pull back the stent deployment grip until the outer X‐ray ring overlaps with the X‐ray marker on the inner sheath. (Figure 2e). Finally, check the opening of the distal flare. Subsequently, the stent deployment grip lock must be unlocked (Figure 2f), and the stent deployment grip to the indicated pinpoint (Figure 3a) to deploy the first flare. For the deployment of the proximal flare, there are two approaches under endoscopic visualization or under EUS guidance depending on endoscopist preference. The two approaches could be used complementary or separately in accordance with EUS or endoscopic view. With this novel device, you could choose one or the other depending on the indication, habits, GI tract, and space as well as the other Hot LAMS already existing.

#### Under endoscopic visualization

After releasing the main catheter lock, the entire delivery system is pulled back until the blue marker of the outer sheath can be seen under endoscopic view and the main catheter must be locked. Then the second flare is ready to deploy by removing the pin and pulling the stent deployment grip all the way back (Figure 3b).

#### Under EUS guidance

With the first flare in EUS view attached to the wall of the targeted organ (retracting the main catheter control), the main catheter should be locked when the first flare approaches the inner wall. After removing the pin the blue handle needs to be completely brought up (Figure 3c) which deploys the proximal flare of the stent (Figure 3d). The second flare of the stent is now released but may remain in the working channel. Finally, the main catheter control should be advanced while retracting the scope in a 1‐to‐1 fashion until the second flare is released from the scope and visualized on endoscopy or EUS.

#### Delivery system removal from echoendoscope

Following the deployment of the stent, the luer lock needs to be unlocked at the base of the handle. This is done by twisting the luer lock in a counterclockwise fashion and removing the delivery catheter by pulling it upward and out of the working channel.

#### Procedure description on porcine models

The Hot‐Spaxus 2 was tested on four pigs (weighing from 43 to 52  kg, respectively). To facilitate EUS scope intubation of the esophagus, an overture was placed. All the pigs were under general anesthesia and cardiopulmonary monitoring after fasting for 12 h. After the completion of experimental interventions and post‐mortem assessments, euthanasia will be carried out using intravenous injection of sodium pentobarbital (100 mg/kg), a barbiturate commonly used for euthanasia in laboratory animals.

The animals will be first sedated, and upon loss of consciousness, the sodium pentobarbital will be administered intravenously. The pigs will be closely monitored to confirm that there was no further physiological activity post‐injection.

EUS‐guided gallbladder drainage (EUS‐GBD) was attempted either from the gastric or duodenal site, depending on the best possible site where the gallbladder could be visualized. Additionally, three EUS‐GJ were planned by placing the LAMS from the stomach into a jejunal loop. All of the procedures were performed with freehand technique and the deployment of the proximal flange was done with the intrachannel release technique, under EUS guidance.^15^ Safety was defined by the rate of AEs. Technical success of LAMS placement was identified by the correct positioning of both flanges, the distal one inside the specific target and the second one in the gastric or duodenal lumen. Additionally, any strengths and weaknesses of the new Hot‐Spaxus 2 were recorded and discussed with the team which consisted of two experienced endosonographers (Benedetto Mangiavillano and Francesco Auriemma), a GI fellow (Gianluca Franchellucci), and Taewoong Medical's engineering team.

All procedures were carried out with a Pentax therapeutic linear echoendoscope (model EG‐3870UTK) and a gastroscope (model EG‐2990i) by an experienced endoscopist (Benedetto Mangiavillano). To distend the gallbladder rendering drainage easier to be performed, a through‐the‐scope clip (TTSC) was first placed at the level of the major papilla with the intention of closing the biliary orifice.

For the EUS‐GJ procedures, a saline solution infused with blue dye was utilized to achieve the necessary expansion of the jejunal loops. Once the target loop was identified, a Doppler examination was conducted to ensure there were no interposed vessels and to determine the optimal puncture site. Access to the loop was then gained utilizing the freehand technique with pure cut (80–120 W and 400–500 Vp applied using the ERBE VIO 300D generator; ERBE USA Inc.).

## RESULTS

In the four pigs, a total of sixteen LAMS were deployed which included four EUS‐GBD procedures and 12 EUS‐GJ procedures. All LAMS were successfully deployed. Four EUS‐GBD were performed (one in each pig) using Hot Spaxus 8 mm diameter × 10 mm length stents. Two procedures were performed using a transgastric approach while the other two procedures were performed using a transduodenal approach. The mean width (on three different measures) between the gallbladder and stomach versus the duodenal bulb was 7.02 ± 0.68 mm versus 7.05 ± 0.79 mm, respectively.

The mean procedure time for EUS‐GBD was 16.15 ± 2.72 min (from intubation with the EUS scope to scope extraction). For EUS‐GJ, 20 × 15 mm Hot Spaxus 2 were placed (12 in total; three for each pig), with a mean procedure time of 23.85 ± 3.41 min. The jejunal loop was injected with water and methylene blue until a minimum diameter of 20 mm was obtained before performing the procedure.

In all procedures, the distal and proximal flanges were safely released without any AEs. There were no issues with the new handle. The physiological parameters of the porcine models remained within the normal range throughout the procedures, and no AEs, according to ASGE lexicon^16^ were reported by the endoscopist or anesthesiologist.

## DISCUSSION

We performed an animal study to test the safety and technical feasibility of the novel Hot‐Spaxus 2. This new stent was modified and enhanced to overcome some of the limitations of the first version. Gallbladder drainage and gastro‐jejunal anastomoses utilizing Hot Spaxus 2 were successfully performed in four pigs, without any procedural or periprocedural AEs.

Hot‐Spaxus 2 placement procedures were performed by a seasoned endosonographer (Benedetto Mangiavillano) with significant experience in LAMS placement, along with a panel of experts (one expert endosonographer, one fellow, and one Taewoong engineer) prior to CE mark submission. The LAMS structure was easily visualized through EUS at each stage of the LAMS release. Despite the porcine stomach being slightly thinner than the human stomach, the LAMS proved to be successful in penetrating the tissue without requiring multiple attempts to reach the target loop in both EUS‐GJ and EUS‐GBD procedures.

In comparing the new handle of the LAMS delivery system to the previous Hot‐Spaxus, it was found to be more comfortable for the single operator to manage both the LAMS release and endoscopic activity during deployment. A crucial step is the post distal flange release when the handle gives a reassuring ‘shot’ to confirm the safe deployment of the flange and prevent accidental release of the proximal one. Four EUS‐GBD procedures were performed, two from the gastric cavity and two from the duodenum, with no observed differences. In this study, we used the freehand technique as this was the preference of the endoscopist. The use of the guide‐wire placed inside the targeted organ with a 19G needle, and subsequent exchanging of the needle with the LAMS, can move the wire with its possible misdeployment. Moreover, the exchange of the needle with the LAMS can modify the penetration axis of the device, disassembling the axis of the stomach or the duodenum with the axis of the targeted organ, conditioning the success of the procedure.

The study has certain limitations. First of all the absence of a direct comparison of this new model of Spaxus stent with other LAMS available on the market, such as the Axios stent (Electrocautery‐enhanced Hot‐Axios; Boston Scientific Corp.). Direct comparison among the two different stents lack for both the EUS‐GBD and EUS‐BD; an indirect comparison was performed in a recent metanalysis^17^ that evidenced that AEs occurred in 23.6% of patients with the AXIOS stent and 9.5% of patients with the SPAXUS stent during EUS‐GB. Furthermore, a direct comparison among the two different stents was performed by our group in pancreatic fluid collection drainage, where we observed that the SPAXUS stent showed fewer bleeding events.^18^ Currently, no direct and indirect comparisons are available for EUS‐GE in our knowledge. It's important to note that the absence of technical failure obtained in the study is due to different elements, such as the fact that all the procedures were performed by high‐skilled endoscopists in LAMS placement. Then, this pilot study used a small sample size with normal animal models and did not consider pathological clinical scenarios in which EUS‐GBD and EUS‐GJ are routinely performed.

Additionally, in the EUS‐GJ procedure in a porcine model, in the absence of stenosis or peritoneal carcinomatosis, the endosonographer can choose the best target loop at every site, regardless of whether it's a jejunal or ileal loop. In some human cases, jejunal stenosis can determine the site of the target loop. Another crucial aspect to consider is the impact of the endoscopist's anxiety level on the technical aspect of procedures. In challenging situations, the operator's anxiety can influence the success of the procedure,^19^, ^20^ whereas in porcine models, this level of stress is likely reduced or absent.

In conclusion, the updated design of the Hot‐Spaxus 2, particularly the handle, has addressed some of the limitations of the first‐generation stent and has resulted in a high‐performance LAMS that can be operated by a single endoscopist. Despite no AEs encountered using porcine models, additional studies are necessary to evaluate the safety and effectiveness of the stent in human patients, and further experiences are needed to compare these new LAMS with the available ones on the other market.

## CONFLICT OF INTEREST STATEMENT

Benedetto Mangiavillano is a consultant for Taewoong; Alberto Larghi is a consultant for Boston Scientific; Alberto Larghi is a consultant for Boston Scientific, Fujifilm, and Medtronic. The other authors declare no conflict of interest.

## ETHICS STATEMENT

The institutional review board of Humanitas Mater Domini approved the study (no. 04/2025 HMD).

## PATIENT CONSENT STATEMENT

N/A

## CLINICAL TRIAL REGISTRATION

N/A
